# Enhancing the Resistive Switching Properties of Transparent HfO_2_-Based Memristor Devices for Reliable Gasistor Applications

**DOI:** 10.3390/s24196382

**Published:** 2024-10-01

**Authors:** Taegi Kim, Doowon Lee, Myoungsu Chae, Kyeong-Heon Kim, Hee-Dong Kim

**Affiliations:** 1 Department of Semiconductor Systems Engineering, Convergence Engineering for Intelligent Drone, Institute of Semiconductor and System IC, Sejong University, 209, Neungdong-ro, Gwangjin-gu, Seoul 05006, Republic of Korea; 2 Division of Electrical, Electronic and Control Engineering, Kongju National University, Cheonan 31080, Republic of Korea; 3 Institute of Industrial Science, University of Tokyo, 4-6-1 Komaba, Meguro City 153-8505, Tokyo, Japan; 4 Department of Convergence Electronic Engineering, Gyeongsang National University, Jinju-si 52725, Republic of Korea

**Keywords:** transparent memristor, gasistor application, resistive switching, roughness of BE

## Abstract

We present a transparent memristor with a rough-surface (RS) bottom electrode (BE) with enhanced performance and reliability for a gasistor, which is a gas sensor plus a memristor, and its application in this paper. The transparent memristor, with an RS BE, exhibited low forming voltages (0.8 V) and a stable resistive switching behavior, with high endurance and an on/off ratio of about 125. This improvement is due to the better control of the electric field distribution and the oxygen vacancy concentration when applying the RS BE to transparent memristors. Maintaining the stability of the conducting filament in an ambient air environment for extended periods of time is crucial for the application of memristors as gasistors. The memristor with an RS BE demonstrates an ability to sustain a stable-current state for approximately 10^4^ s. As a result, it is shown that the proposed transparent memristor with an RS BE can significantly enhance the device’s reliability for gasistor applications.

## 1. Introduction

**Figure 1:**
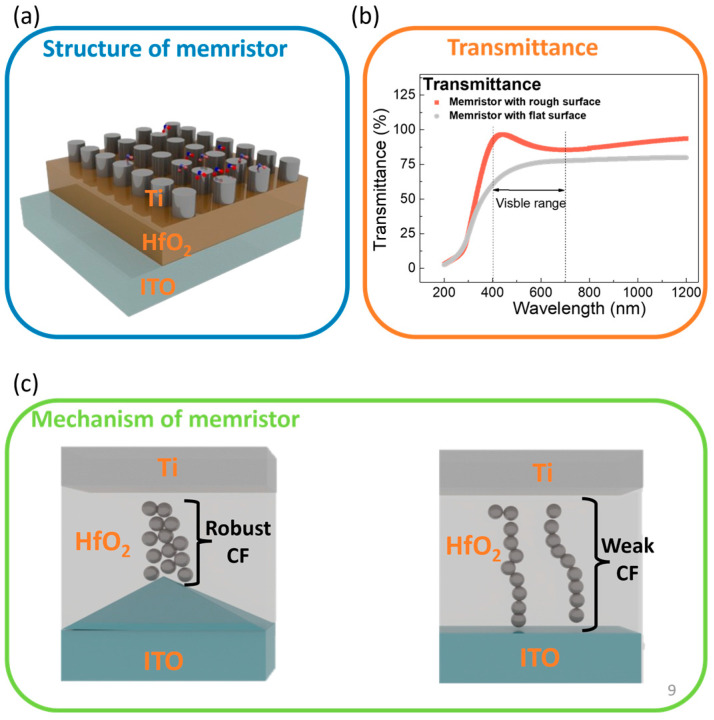
(**a**) Schematic of a transparent memristor that illustrates the effect of BE surface roughness on the robustness of CF within the transparent memristor structure. (**b**) Transmittance of a transparent memristor with and without an RS BE. (**c**) Cross-sectional schematic representation that illustrates memristors with an FS BE and RS BE.

Transparent memristors are gaining a significant amount of attention in the field of transparent electronic applications, such as in data storage and processing [1,2,3,4]. HfO_2_-based memristors are, in particular, gaining attention due to their transparency and low power consumption, which makes them ideal for next-generation transparent electronics [5]. Recently, these devices have been adapted for gas sensing applications, known as gasistors, which are a hybrid of a gas sensor and memristor. Gasistors exhibit exceptional gas sensing capabilities, including high sensitivity at room temperature (RT), excellent selectivity for detecting mixed gasses, and rapid recovery when using pulse biasing. The combination of a memristor and a gas sensor offers significant advantages by enhancing their gas detection performance and maintaining their low energy consumption, which makes them highly suitable for a wide range of practical applications. This research provides valuable insights into optimizing gasistor performance, especially in environments where precise and efficient gas detection is critical [6,7,8,9,10]. The operation of memristors in a gasistor critically depends on the precise control of the conductive filament (CF), which is essential for accurate gas detection [9]. Previous studies demonstrate that roughening the surface of the bottom electrode (BE) through etching processes can stabilize the control of the CF, which is vital for reducing variability in set/reset voltages and currents, which enhance the device’s reliability [11,12,13]. However, conventional etching processes often introduce environmental concerns due to the generation of waste [14]. Recent studies have therefore focused on improving the surface roughness of the BE by increasing the deposition temperature [13,15]. We propose a transparent memristor with a rough surface (RS) BE in this study, which is shown in Figure 1a. We optimized the roughness of the BE by adjusting the sub-heater temperatures during the deposition process to avoid additional post-processing. This method leads to improved performance characteristics for transparent memristors used in gasistor applications, which include low fluctuations in the set/reset voltage and a high on/off ratio, which are crucial for reliable and precise gas detection. The application of memristors in a gasistor requires these components to exhibit stable resistive switching properties and maintain functionality under low operational voltages. The large ratio between the high-resistance state (HRS) and the low-resistance state (LRS) also mitigates issues such as read errors by enhancing the device’s reliability even after numerous operations. Furthermore, the CF must demonstrate long-term stability in an ambient atmosphere in order to ensure continuous and reliable interaction with target gasses. As a result, the proposed device, with an RS BE, shows improved reliability attributes, such as its on/off ratio, retention, power consumption, and endurance, and it also demonstrates a significant increase in transmittance, features which are displayed in Figure 1b,c. The transmittance in the visible spectrum of a memristor with an RS BE specifically reaches 83%, which is approximately 10% higher than that of devices without an RS BE. Our study contributes to advancing the technology behind transparent memristors and expands their application spectrum in the field of gas detection by elucidating the impact of an RS BE on their resistive switching properties.

## 2. Materials and Methods

### 2.1. Fabrication Process of the Transparent Memristor

The fabrication process was conducted using five different structures in order to compare the switching characteristics of the memory devices. First, indium tin oxide (ITO) was deposited at various temperatures for an flat-surface (FS) device using RF sputtering (KVS-2000L), and its sheet resistance was measured using a four-point probe (AIT, CMT-SR2000N) with a sheet resistance accuracy of ±0.5%. The patterned ITO was fabricated by varying the sub-heater temperature during RF sputtering in order to create structures with surface roughness. Flat structures deposited at room temperature were subjected to rapid thermal annealing (RTA, ULVAC, MILA-5000) at 500 °C for 60 s in a N_2_ atmosphere in order to match the sheet resistance of the samples deposited at 500 °C as a control. The other samples were, in contrast, deposited at sub-heater temperatures that ranged from 200 to 500 °C, without additional heat treatments. A 25 nm thick HfO_2_ insulating layer was subsequently deposited via ALD at 280 °C. Finally, a 50 nm Ti top electrode was deposited at RT using RF sputtering, as is shown in Figure 1a.

### 2.2. Characterization of the Transparent Memristor

The optical transmittance of the trans with and without RS BE structures was obtained using a UV–vis spectrophotometer (Cary 5000 UV–vis–NIR spectrophotometer, Agilent Technologies Inc., Santa Clara, CA, USA) with a wavelength accuracy of ±0.1 nm in a spectral range from 300 to 1100 nm. We additionally measured the ITO BE surface roughness depending on the sub-heater temperature by using Atomic Force Microscopy (AFM, Park System Corp, XE-100, suwon city, Gyeonggi-do, Republic of Korea) in order to observe its morphology.

### 2.3. I–V Characterization of the Transparent Memristor

The current–voltage (I–V) characteristics of the fabricated memristor were evaluated. The memristor device was placed inside a dark box in order to minimize external influences. The top electrode (TE) was connected to a Keithley 4200 SCS (current: ±0.025%, voltage: ±0.012%), whereas the BE was grounded. The I-V curves of the proposed memristor were analyzed by performing DC voltage sweeps. Additional procedures were conducted to assess the state of the CF beneath the TE in order to check for any changes following the I-V curve measurements. The I–V characteristics of the memristor were comprehensively evaluated via these steps.

### 2.4. Electrical and Gas Sensing Characterization of Gasistor

The chamber temperature was maintained at RT and the humidity was maintained at 20% relative humidity (RH). The total gas flow rate was fixed at 500 sccm. O_2_ (99.99%) gas was injected into the chamber for approximately 50 s before the nitric dioxide (NO_2_) gas injection. The NO_2_ gas was then injected and O_2_ was used as the carrier gas. NO_2_ gas was injected continuously to evaluate the response characteristics of the gas sensor. In addition, for practical applications, experiments were conducted to evaluate the gasistor’s response characteristics using air (99.999%) as a balance gas. To evaluate the gas sensing characteristics of the gasistor depending on the BE’s surface roughness, its transient response was monitored at a sensing voltage of 0.4 V. Gas sensing tests were also performed using a 30 cm^3^ aluminum chamber isolated from the external environment. The dry O_2_ gas used in this system was 99.99% pure. The NO_2_ gas concentration was set at 50 ppm and air was used to balance the mixture. The chamber temperature was kept at RT and the humidity was maintained at 20% relative humidity (RH). In addition, the total gas flow rate was fixed at 500 sccm. After the above initialization, the chamber was flushed with O_2_ for 100 s to clean the chamber before the injection of NO_2_ gas and dry O_2_ carrier gas. The target gas and carrier gas were then injected into the chamber to evaluate the response characteristics. The target gas and O_2_ were allowed to flow until there was no further change in current and were allowed to react with the gasistor. The concentration of NO_2_ gas was maintained at 25 ppm via a mass flow controller (MFC; DFPC1000, Daejeon city, Republic of Korea). The flow rate of dry O_2_ and NO_2_ was varied while keeping the total flow rate at 500 sccm to determine the response characteristics as a function of concentration. In order to assess the response, we converted the measured current to the response using Equation (1), where I_air_ and I_gas_ represent the current levels before and after the NO_2_ exposure, respectively, under dry air injection.

(1) $$\mathrm{Response}\;(\%)=\frac{I_{gas}-I_{air}}{I_{air}}\times 100$$

## 3. Results and Discussion

### 3.1. Material Characteristics of RS BE

**Figure 2:**
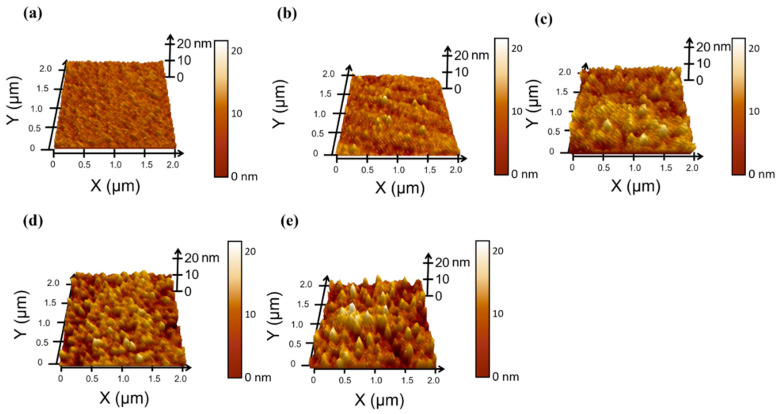
AFM topology images of ITO deposited at different sub-heater temperatures of (**a**) RT, (**b**) 200, (**c**) 300, (**d**) 400, and (**e**) 500 °C.

The crystallinity and roughness of the BE were controlled by varying the sub-heater temperature in order to optimize the surface roughness characteristics of the ITO films used for the memristor. AFM measurements were conducted in order to objectively analyze the surface morphology of the ITO. Figure 2 and Table 1 present the topography images and the measured values of its average roughness (R_a_), root mean square roughness (R_q_), and peak-to-valley roughness (R_pv_) within a 2 × 2 µm^2^ area. As the sub-heater temperature increased from RT to 500 °C, the R_a_, R_q_, and R_pv_ of ITO all showed an increasing trend. FS and RS 200 in particular exhibited smooth surfaces, with R_q_ values of 1.48 and 1.53 nm, respectively. The R_q_ of RS 400 notably increased from 1.68 to 2.32 nm, which indicates the onset of ITO crystallization. ITO deposited at higher sub-heater temperatures showed increased density and roughness, and RS 500 exhibited the highest surface roughness at an R_q_ of 3.23 nm, which is in addition to an apparent increase in the grain size. These observations are consistent with the findings from previous studies [15,16].

### 3.2. Operation Priciple of a Low-Power Memristor with RS BE

**Figure 3:**
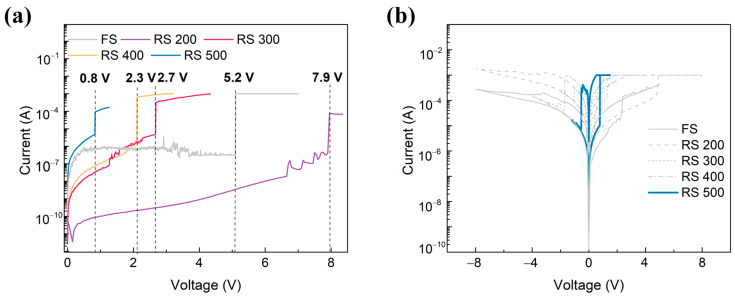
Resistive switching characteristics of FS, RS 200, 300, 400, and 500. (**a**) The forming process and (**b**) the I–V curve, depending on the surface roughness of the BE.

The I-V curves in DC mode were measured in order to analyze the RS characteristics of HfO_2_-based memristors according to the roughness of their ITO BE. The samples designated as RS 200, RS 300, RS 400, and RS 500 were named according to the sub-heater temperatures of 200, 300, 400, and 500 °C, respectively, used during the ITO deposition process. The FS sample was prepared in order to have the same sheet resistance as RS 500 for a more accurate comparative analysis regarding their roughness. ITO was deposited in RT sub-heater conditions and then subjected to RTA at 500 °C in a N_2_ atmosphere by adjusting the sheet resistance to 23 Ω/□ in order to achieve this. A forming process was applied to break down the dielectric layer at approximately the forming voltage before normal operation in the bipolar resistive switching mode. The current increased sharply when the forming voltage was applied, because oxygen vacancies were created within the dielectric layer. The application of the forming voltage generated a strong electric field, which drives oxygen ions away from the dielectric and leaves behind oxygen vacancies [17]. These vacancies aggregated to CFs, resulting in the transition from an HRS to an LRS. Figure 3a shows that the I-V measurements were conducted under a 1 mA compliance current for the FS, RS 200, RS 300, RS 400, and RS 500 devices, which transitioned from an HRS to an LRS. Each forming voltage exhibited was as follows: 5.2, 7.9, 2.7, 2.3, and 0.8 V. RS 200 showed a comparatively higher forming voltage than FS, which is likely due to the high sheet resistance of the BE. However, RS 300, 400, and 500 exhibited decreased forming voltages compared to FS, and RS 500 had the lowest, at 0.7 V. The decrease in the forming voltage with increasing surface roughness aligns with previous studies [11,12]. During the reset process, where FS, RS 200, RS 300, RS 400, and RS 500 were reset at −8.14, −7.6, −1.1, −1.7, and −0.53 V, respectively, the CFs are partially or fully ruptured, causing the devices to return to an HRS. This occurs because the reverse electric field drives oxygen ions back into the vacancy sites, disrupting the CFs. The lower reset voltages for RS 300, RS 400, and RS 500 are attributed to their rougher BE surfaces, which enhance the local electric field at the vertex, making it easier for oxygen ions to redistribute and break the CFs [18]. In the set process, the devices were set to 2.4, 4.9, 1.3, 0.5, and 0.77 V, respectively. Here, oxygen vacancies are reintroduced into the dielectric under a forward bias, which reforms the CFs and switches the devices back to an LRS. The reduction in the set voltage for a memristor with an RS BE is attributed to the higher local electric fields at the BE vertex, which facilitate the re-formation of CFs at low voltages.

### 3.3. Reliability of a Memristor with an RS BE

**Figure 4:**
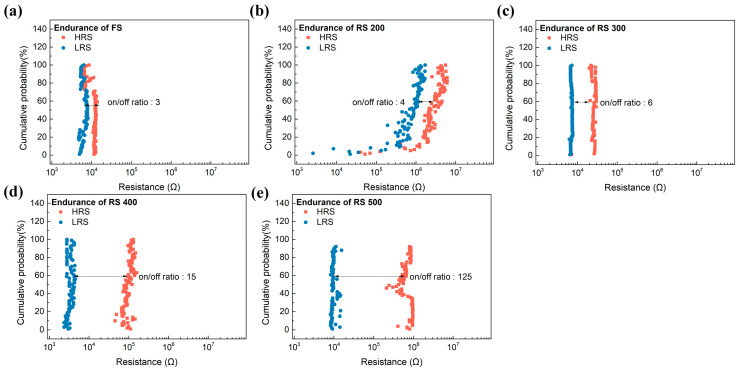
Endurance of the memristor depending on the BE’s roughness. (**a**) FS, (**b**) RS 200, (**c**) RS 300, (**d**) RS 400, and (**e**) RS 500.

Figure 4 shows the endurance of the memristor as a function of the surface roughness over 100 cycles in the DC bias mode, from which the on/off ratio was determined. The current ratio (CR) for FS, RS 200, RS 300, RS 400, and RS 500 was observed to be 3, 4, 6, 40, and 125, respectively. This trend indicates that as the roughness of the BE increases, the on/off ratio of the memristor also increases. This phenomenon occurs because the local electric field that is applied to the BE becomes stronger as the roughness of the BE increases [19]. This intensified electric field lowers the energy barrier that is required for the formation and removal of the oxygen vacancy, which are crucial with regard to creating CFs [20]. The lower energy barrier facilitates the formation of more well defined and stable CFs. These robust CFs reduce the variability in their formation, leading to a consistent and uniform resistive state. The on/off ratio consequently increases, because a more uniform resistive state ensures that the resistance levels in the LRS and the HRS are well defined and distinct. The CR between the LRS and the HRS becomes more pronounced, which leads to a larger on/off ratio and the enhanced robustness and uniformity of the CFs [21]. When a rough-surface BE was used, the CR was at least 41 times higher than that of the sample without an RS BE, which means that an increase in response might be possible [8].

**Figure 5:**
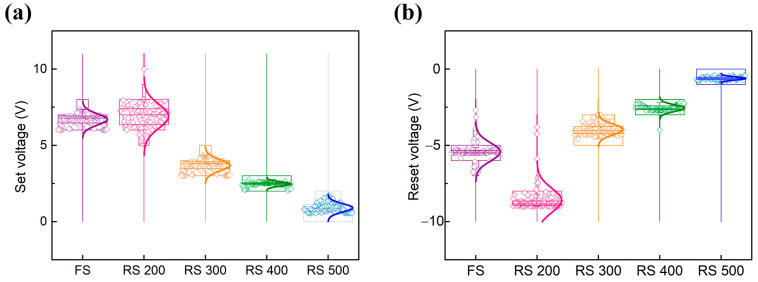
(**a**) Set and (**b**) reset voltage distributions of the memristor depending on the surface roughness of its BE.

The distribution of the set/reset voltage plays a critical role with regard to determining the gas sensing performance of a gasistor, because a stable voltage distribution enhances the reproducibility of the gasistor, which ensures consistent results across multiple measurements of the same gas concentration [8]. These voltages are crucial for transitioning the device between its LRS and HRS. A broad distribution of these voltages necessitates higher applied voltages for reliable switching, because low set voltages may fail to induce the transition. This requirement leads to increased power consumption and reduced device endurance, which adversely affects the reliability of memristors over time [22]. Figure 5a,b show that we evaluated the distribution of the set/reset voltages for FS, RS 200, RS 300, RS 400, and RS 500. The results indicate a trend where the distribution narrows by increasing the surface roughness of the BE. The standard deviation of the set voltage is notably a critical factor. FS and RS 200 exhibited standard deviations of 0.47 and 0.46 in their HRS, respectively, which indicates operational variability and potential performance inconsistency. However, as the surface roughness of the BE increased, the standard deviation decreased, and RS 500 demonstrated a standard deviation of 0.13 in its HRS. This reduction is attributed to the improved local electric field uniformity that was achieved by increasing the BE surface roughness, which thereby enhanced the uniformity of the set/reset voltages [23]. These phenomena suggest that a rougher BE surface is advantageous for gasistor applications, as it offers low power consumption and high reliability [20].

**Figure 6:**
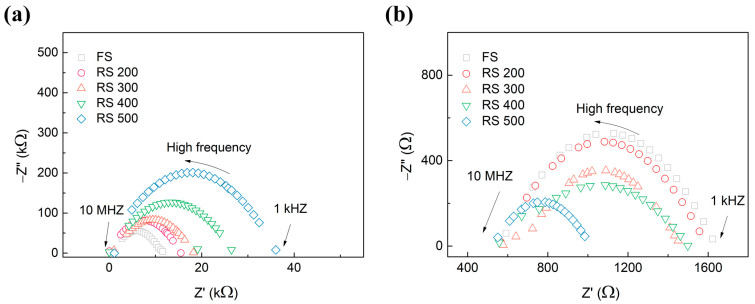
Impedance spectroscopy of FS, RS 200, 300, 400, and 500 in (**a**) the HRS and (**b**) the LRS.

Impedance measurements were conducted in order to investigate the cause of the increased stability seen within the resistive switching phenomena as the surface roughness of the BE increases. Figure 6a,b show the Nyquist plots of the memristor at its LRS and HRS, respectively. The Nyquist plots corresponding to the various roughness levels of the BE, which included FS, RS 200, RS 300, RS 400, and RS 500, were effectively fitted using Equation (2). The impedance of the equivalent circuit can be described using the equation that is provided below.

(2) $$Z\left(w\right)=R_{f}+R_{c}+\frac{R_{p}}{1+{(wR_{p}C_{p})}^{2}}-j\frac{wR_{p}^{2}C_{p}}{{1+(wR_{p}C_{p})}^{2}}$$

where Z is the complex impedance, ω is the angular frequency, R_f_ and R_c_ are the equivalent series resistances of the residual CF and measurement connections, respectively, and R_p_ and C_p_ are the equivalent parallel resistance and capacitance of the leakage gap, respectively [24]. The series resistance (R_s_) is the sum of the resistance of the residual filament and the contact resistance. However, the contact resistance can be neglected due to its significantly lower value compared to the resistance of the filament [24,25]. The values of the components in the equivalent circuit are shown in Table 2. The R_s_ and R_p_ values show an obvious decreasing trend when the surface roughness of BE changes from FS to RS 500 in its LRS. However, it has been observed that the R_s_ and the R_p_ values increase in the HRS as the surface roughness of the BE increases. The observed phenomena can be attributed to its increased ability to selectively adjust the CF as the roughness of the BE increases [12,26]. This leads to improvements in the memristor’s performance by enabling the more precise and rapid detection of the target gas.

**Figure 7:**
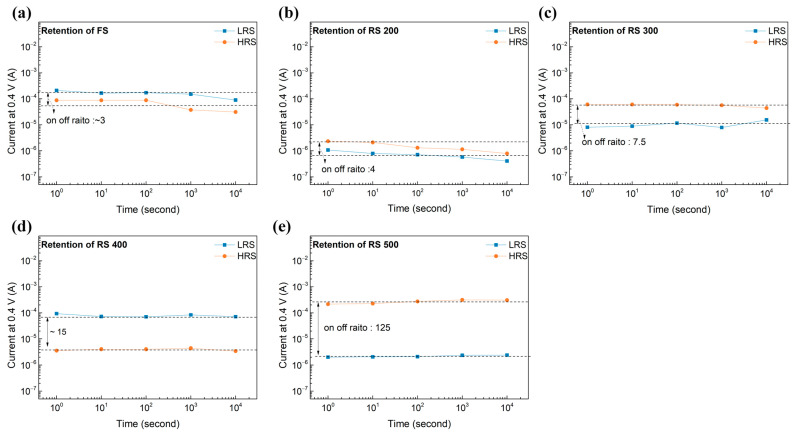
Retention of the memristor depending on the BE’s surface roughness. (**a**) FS, (**b**) RS 200, (**c**) RS 300, (**d**) RS 400, and (**e**) RS 500.

We measured the current state of the transparent memristor in its LRS and HRS for 10^4^ s at a V_read_ of 0.4 V in order to determine whether the CFs were maintained in ambient air. This V_read_ was selected because it shows the highest on/off ratio, which thereby enhances the reliability of gas detection. Figure 7 illustrates that the retention characteristics were verified by measuring the LRS and the HRS of the FS, RS 200, 300, 400, and 500 for 10^4^ s in order to ensure that the CF remained deformation-free at a V_read_ of 0.4 V. All memristors exhibited 10^4^ s of a reliable resistive state except for the FS and RS 200, which had a low surface roughness to their BE. This phenomenon can be attributed to the relationship between their defects and the retention time. Previous studies show that retention properties improve by reducing the defects around the filaments [27,28]. The improved retention properties of RS 500 can consequently be explained by the reduced generation of defects around the filaments.

Table 3 presents a performance comparison of HfO_2_-based memristors. Most existing transparent memristors still rely on a conventional FS BE, which is due to its reliable and durable operation in controlled environments. However, memristors with FS BE often encounter high forming voltages and limited stability, which increase their overall energy consumption and hinder their long-term reliability in gasistor applications. Ongoing research aims to optimize the design and materials of the BE in order to enhance their performance, but challenges remain with regard to achieving consistent switching behavior and low power operation. The performance of the proposed transparent memristor with an RS BE, in contrast, demonstrated superior characteristics, which were verified by our experimental results. This is attributed to the improved electric field distribution and better control over the oxygen vacancy concentrations that were enabled by the RS BE. Moreover, retention tests confirmed that the device maintains stable resistive switching for up to 10^4^ s in ambient air, which validated its enhanced reliability and suitability for long-term gasistor applications.

**Figure 8:**
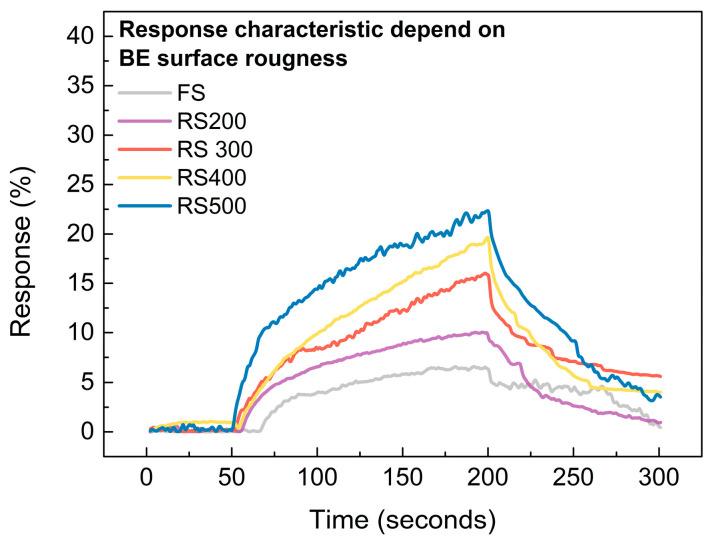
Transient response characteristic of FS and RS 200, 300, 400, and 500 devices, depends on their BE surface roughness, in 25 ppm NO_2_ gas.

To investigate the memristors’ gas response characteristics based on their BE surface roughness, 25 ppm of NO_2_ gas was injected for 150 s, and their current variation was measured at a read voltage of 0.4 V. Figure 8 illustrates that it was observed that the response rate increased as the NO_2_ injection time increased. This phenomenon was due to the adsorbed NO_2_ gas near the CFs of the HfO_2_-based gasistor being able to lower the Schottky barrier height between the CFs and the TE, which corroborates the findings of a previous study [33,34]. Notably, the RS device exhibited a response of 22.44%, whereas the RS 500 device showed a 6.43% response, which represents a 249% increase compared to the FS device. This behavior is attributed to its increased BE surface roughness, which stabilizes the CF and leads to more reliable gas detection.

## 4. Discussion

It is essential to achieve precise control of the CF, ensure a large on/off ratio between the HRS and the LRS, maintain low power consumption, and exhibit high retention properties in order to effectively using memristors in gasistor applications. Our proposed device concentrates the electric field over the RS BE by facilitating the formation of a stable CF at a low voltage of 0.8 V. This is crucial because stable CFs ensure precise control over resistive switching, which is essential for accurate gas detection. The memristor exhibits high on/off ratios, which reach up to 125, and a narrow distribution of set/reset voltages, which are necessary for maintaining consistent and reliable gas sensor operation under varying environmental conditions. Memristors with an RS BE also demonstrate superior endurance, maintain their performance over 100 cycles, and have retention capabilities that exceed 10^4^ s. These attributes are important with regard to ensuring long-term reliability and reducing power consumption, which makes them suitable for continuous and efficient gas sensing. An optimized memristor with an RS BE is well suited for gasistor applications due to its enhanced resistive switching characteristics, power efficiency, and reliability. This comprehensive approach addresses the need for precise, low-power, and reliable gas detection, which is highlighted in the introduction. This is vital in terms of expanding the use of memristors in transparent electronic applications.

## 5. Conclusions

Optimizing the roughness of the BE in transparent memristors significantly enhances their performance in gasistor applications, as demonstrated in this study. We achieved a higher BE roughness, which resulted in more pronounced resistive switching characteristics, by increasing the sub-heater temperature during the deposition process. The rough surface of the BE concentrated the electric field at the apexes, which facilitated the formation of a robust CF at 0.8 V. This led to improved memristor endurance, which is proven by a higher on/off ratio and reduced variability in the set/reset voltages. Impedance spectroscopy and retention measurements further confirmed the enhanced stability and reliability of memristors with an RS BE. To further verify that their gas reaction characteristics increase with the increase in their BE surface roughness, gas sensing experiments were conducted. It was confirmed that the RS 500 device exhibited a response characteristic of 22.4% to 25 ppm of NO_2_ gas. It can be utilized as a foundational element for a gasistor that possesses the high integration and low power consumption attributes that are essential in advanced industries.

## Figures and Tables

**Table 1:** AFM measurements of R_a_, R_q_, and R_pv_ of ITO BE formed at different sub-heater temperatures.

Sub-Heater Temperature (°C)	Roughness (nm)		
	R_a_	R_q_	R_pv_
RT	1.18	1.48	10.32
200	1.21	1.53	10.48
300	1.24	1.68	12.38
400	1.67	2.32	21.23
500	2.41	3.23	28.31

**Table 2:** The values of the components in an equivalent circuit for the LRS and the HRS states of the memristor, depending on the surface roughness of its BE.

State	Parameter	FS	RS 200	RS 300	RS 400	RS 500
HRS	R_s_ (Ω)	592	565	558	541	523
	R_p_ (kΩ)	1.03	0.991	0.873	0.836	0.434
	C_p_ (pF)	4.31	4.47	4.82	5.29	8.26
LRS	R_s_ (Ω)	631	654	662	685	693
	R_p_ (kΩ)	10.7	13.7	18.2	26.4	35.4
	C_p_ (nF)	14.3	9.26	7.83	3.63	1.85

**Table 3:** Comparison of HfO_2_-based memristors’ performance.

Device Structure	Parameter					
	V_Forming_ (V)	V_set_ (V)	V_reset_ (V)	On/Off Ratio	Retention (s)	Ref.
Pt/HfO_2_/TiO_2_/HfO_2_/Pt	-	4	−2.5	100	10^4^	[29]
CNT/HfO_2_/Pt	10.95	7.9	−3.95	100	10^4^	[8]
Ti/HfO_2_/Pt	-	0.88	−0.89	11.4	10^4^	[30]
Pt/HfO_2_/TiO_2_/HfO_2_/Pt	3.2	1.5	−0.5	<100	10^4^	[31]
Pt/HfO_2_/TiO_2_/ITO	3.1	1.6	−1.5	10	10^4^	[32]
Ti/HfO_2_/ITO with RS BE	0.8	0.77	−0.53	125	10^4^	In this work

## Data Availability

The data are contained within the article.
